# Evolution and Function of the Chloroplast. Current Investigations and Perspectives

**DOI:** 10.3390/ijms19103095

**Published:** 2018-10-10

**Authors:** Bartolomé Sabater

**Affiliations:** Department of Life Sciences (Ciencias de la Vida), University of Alcalá, Alcalá de Henares, 28805 Madrid, Spain; bartolome.sabater@uah.es; Tel.: +34-609-227-010

Chloroplasts are the place for the major conversion of the sun’s radiation energy to chemical energy that is usable by organisms. Accordingly, they account for about 50% of the leaf protein [1], and the enzyme ribulose-1,5-bisphosphate carboxylase of chloroplast is by far the most abundant protein on the Earth [2]. Chloroplasts are not only key in photosynthesis, they are the place of the synthesis of fatty acids in plants and biosynthesis of amino acids, porphyrins, isoprenoids, and secondary metabolites that, sometimes, duplicate parallel biosynthetic pathways in cytosol. Only a small fraction of the involved enzymatic machinery is encoded in the chloroplast DNA. Nuclear DNA encodes most of the chloroplast proteins which, after being synthesised as precursors on cytosol ribosomes, are incorporated into the appropriate chloroplast substructures. Chloroplast evolutionarily derives from a primitive cyanobacteria that was engulfed by non-photosynthetic cells and, progressively, after losing most of its DNA, became the actual chloroplast that retains only a fraction of the original cyanobacterial genes. Most of the original enzyme machinery is now encoded in the cell nuclear DNA that controls the plastid divisions, among others [3].

The structure and dynamics of the chloroplast photosynthesis machinery is a central focus of molecular investigations on photosynthetic productivity and leaf senescence. Functional coordination among chloroplasts, cytosol, nucleus, and other subcellular compartments inspire many active research topics of chloroplast molecular biology. The transition from the engulfed autonomous cyanobacteria to a non-autonomous endosymbiont chloroplast requires new coordination signals, specific for the different plant cells, which have been actively investigated. In fact, the leaf’s green chloroplasts are members of the plastid organelles present in all plant cells. All plastids share the same DNA and a few structural features and functions (as the synthesis of fatty acids) and derive from the proplastids present in meristematic cells. The biogenesis of the different plastids, their interconversion and the molecular bases of the functions of some plastids (amiloplasts, chromoplasts, etc.) have also been actively investigated at the molecular level. Obviously, only a few research fields may be reflected in this issue which should be the seed for future issues of a broader scope.

Although most of the original cyanobacterial genes have been lost early in the transition to the endosymbiotic chloroplast, a few of them, like the *ndh* genes [4], have been lost later, in some plant lines, endowing characteristic DNA features to specific plant orders, families, genus, or species.

Therefore, the comparison of the sequences of the chloroplast DNA of different plants, provides valuable information on gene content, reordering in the circular chloroplast DNA and mutational genetic-derive, relevant to the evolution of chloroplast and its relations with ancient environments. Increasing facilities for intense genome sequencing have prompted many laboratories to focus on the chloroplast DNA. Reflecting these efforts, more than half of the articles on this issue deal with the functional or evolutionary investigations, based on sequence analyses of chloroplast DNA. Mainly focussed on phylogenetic comparisons of medicinal plant families, articles on *Aristolochia* [5], *Forsythia* [6], *Eucommia* [7], and the *Stemonaceae* family [8] report and analyse the complete sequences of the corresponding chloroplast DNA. Similar approaches on other economically important plants have been reported in articles on genera *Catha* [9], *Primula* [10], *Caragana* [11], *Genciana* [12], and *Quercus* [13], and on the families of *Balsaminaceae* [14], *Meliaceae* [15], *Orchidaceae* [16], and *Fabaceae* [17]. The chloroplast DNA sequence and the analysis of the species of plant branches, that are still poorly investigated have been reported in articles on the Antarctic Bryophyte *Sanionia uncinate* [18], on *Ailanthus altissima* [19], and on genus *Urophysa* [20].

Adding to the sequences reported elsewhere, the chloroplast DNA sequences described in this issue provide a background for precise bar codes that are useful in plant trade and medicinal applications. In addition, chloroplast DNA sequences are excellent tools for taxonomy and phylogeny. Thus, detailed comparison of the chloroplast DNA sequences of the species of *Arabis* [21] led the authors to propose recent events of the interspecific chloroplast capture and hybridization. Similarly, the comparison of the chloroplast DNA sequences of species of the genus *Dendrobium* led authors to identify the mutational biases affecting the GC (guanine + cytosine) content [22]. Not closely related to the chloroplast title of the issue, but potentially useful for rapid comparison of sequences, is the brief article describing a genome-skimming approach in the chloroplast DNA [23].

Pointing to selective pressures reflected in the chloroplast DNA sequence, the comparison of chloroplast DNAs of *Quercus* and some related species led the authors to propose the intriguing possibility that the specific sequences of the *atpF* gene, encoding a subunit of the thylakoid ATP synthase, show significant positive selection for evergreen sclerophyllous oak species, when compared to the deciduous oak species [24].

Chloroplast gene expression is under a complex post-transcriptional control involving C (cytosine) to U (uracil) editing, intron and polycistron splicing and ribosomal activity [25,26]. Transcriptome analysis of the chloroplast genome is reported by Wu et al. Accordingly, the profile of the chloroplast mRNAs is used to identify genes responsible for wheat yellow leaf colour [27]. Approaching, specifically, the question of the post-transcriptional control of gene expression in chloroplasts, the article by Legen and Schmitz-Linneweber reports that a fraction of the chloroplast mRNAs, associated with ribosomes, are attached to the chloroplast as a direct consequence of translation. Plastid mRNA distribution is stable for different plastid types, enabling rapid chloroplast translation in any plastid type [28].

As pointed above, most chloroplast proteins are encoded in the nucleus. The importation of the nuclear-encoded proteins into chloroplasts is a complex process requiring, among others, the recognition of specific sequences in the amino-ends of the precursor proteins that direct them to the appropriate chloroplast substructure. The amino-end sequence of the amino acids required for chloroplast processing has been investigated for the pre-thylakoid rhodanase-like protein (TROL) and has been reported in this issue. Some researchers have combined the site-directed mutagenesis and *in vitro* reconstructed translation and incorporation systems, in an attempt to clarify the dual localization determinants of this protein [29].

Exposed to the changing environmental conditions, functions in chloroplasts are preserved by adapting their metabolism and molecular structures. The article by Shao et al. [30] reports how nitric oxide (NO) protects the photosynthetic apparatus, against water deficit, by affecting the phosphorylation of diverse proteins, among them some are from the primary reaction centre. In their review article, Wang et al. have revised the actual knowledge on the metabolic adaptation of chloroplasts under heat stress [31]. The turnover of protein is a key to the adaptation of chloroplasts to changing conditions. The influence of the extra-plastidic processes on the turnover of chloroplast proteins is analysed in a review article by Izumi and Nakamura [32].

Present knowledge of the mechanisms of the chloroplast division is updated in two review articles [33,34]. The review by Yoshida dealt with the structure and function of supramolecular machinery involved in the plastid division and its resemblance with the mitochondrial division machinery [33]. The review by Irieda and Shiomi updated the knowledge of the mechanism of chloroplast division, as deduced from the heterologous expression systems [34].

Suetsugu and Wada described, in a brief report, the molecular mechanisms controlling the movement of chloroplasts [35]. They explain how two coiled-coil proteins suppress the chloroplast accumulation response mediated by two phototropin-interacting proteins.

The abundance of chloroplast DNA sequence articles in the issue reflects the predominant research projects in the last few years. Certainly, a refinement of the taxonomy and phylogeny investigations requires high numbers of chloroplast DNA sequences to observe the evolutionary trends. Wide analyses of the DNA sequences reported, surely, mark the starting references for further comparisons which could be useful in phylogeny and trade-vegetable characterisation.

Chloroplast DNA articles do not shadow the variety of chloroplast molecular projects, as shown by the other articles on the issue. New technologies have far surpassed the methods and objectives collected in the pioneering methods book, thirty-five years ago [36].

In the coming months, the *IJMS* (*International Journal of Molecular Science*) will certainly include a number of articles dealing with chloroplasts. Many of the articles in this issue have resulted from collaborations of different laboratories, frequently of different countries, which indicates the universality of the field and the necessity of cooperation among specialised research groups, to attack the complex understanding of chloroplasts, at the molecular level. The key role of chloroplasts in the conversion of radiant energy suggests that basic advances in the field would improve plant productivity and environmental protection.

## References

[1] Peoples M.B., Dalling M.J., Noodén L.D., Leopold A.C. (1988). The interplay between proteolysis and amino acid metabolism during senescence and nitrogen reallocation. Senescence and Aging in Plants.

[2] Wildman S.G. (1979). Aspects of Fraction I protein evolution. Arch. Biochem. Biophys..

[3] Martin W., Herrmann R.G. (1998). Gene transfer from organelles to nucleus: How much, what happens, and why?. Plant Physiol..

[4] Martín M., Sabater B. (2010). Plastid *ndh* genes in plat evolution. Plant Physiol. Biochem..

[5] Zhou J., Chen X., Cui Y., Sun W., Li Y., Wang Y., Song J., Yao H. (2017). Molecular structure and phylogenetic analyses of complete chloroplast genomes of two *Aristolochia* medicinal species. Int. J. Mol. Sci..

[6] Wang W., Yu H., Wang J., Lei W., Gao J., Qiu X., Wang J. (2017). The complete chloroplast genome sequences of the medicinal plant *Forsythia suspensa* (Oleaceae). Int. J. Mol. Sci..

[7] Wang W., Chen S., Zhang X. (2018). Whole-genome comparison reveals heterogeneous divergence and mutation hotspots in chloroplast genome of *Eucommia ulmoides* Oliver. Int. J. Mol. Sci..

[8] Lu Q., Ye W., Lu R., Xu W., Qiu Y. (2018). Phylogenomic and comparative analyses of complete plastomes of *Croomia* and *Stemona* (Stemonaceae). Int. J. Mol. Sci..

[9] Gu C., Tembrock L.R., Zheng S., Wu Z. (2018). The complete chloroplast genome of *Catha edulis*: A comparative analysis of genome features with related species. Int. J. Mol. Sci..

[10] Ren T., Yang Y., Zhou T., Liu Z.-L. (2018). Comparative plastid genomes of *Primula* species: Sequence divergence and phylogenetic relationships. Int. J. Mol. Sci..

[11] Jiang M., Haimei M.J., He S., Wang L., Chen A.J., Liu C. (2018). Sequencing, characterization, and comparative analyses of the plastome of *Caragana rosea* var. rosea. Int. J. Mol. Sci..

[12] Zhou T., Wang J., Jia Y., Li W., Xu F., Wang X. (2018). Comparative chloroplast genome analyses of species in Gentiana section *Cruciata* (Gentianaceae) and the development of authentication markers. Int. J. Mol. Sci..

[13] Li X., Li Y., Zang M., Li M., Fang Y. (2018). Complete chloroplast genome sequence and phylogenetic analysis of *Quercus acutissima*. Int. J. Mol. Sci..

[14] Li Z.-Z., Saina J.K., Gichira A.W., Kyalo C.M., Wang Q.-F., Chen J.-M. (2018). Comparative genomics of the balsaminaceae sister genera *Hydrocera triflora* and *Impatiens pinfanensis*. Int. J. Mol. Sci..

[15] Mader M., Pakull B., Blanc-Jolivet C., Paulini-Drewes M., Bouda Z.H.-N., Degen B., Small I., Kersten B. (2018). Complete chloroplast genome sequences of four Meliaceae species and comparative analyses. Int. J. Mol. Sci..

[16] Dong W.-L., Wang R.-N., Zhang N.-Y., Fan W.-B., Fang M.-F., Li Z.-H. (2018). Molecular evolution of chloroplast genomes of orchid species: Insights into phylogenetic relationship and adaptive evolution. Int. J. Mol. Sci..

[17] Liu W., Kong H., Zhou J., Fritsch P.W., Hao G., Gong W. (2018). Complete chloroplast genome of *Cercis chuniana* (Fabaceae) with structural and genetic comparison to six species in Caesalpinioideae. Int. J. Mol. Sci..

[18] Park M., Park H., Lee H., Lee B., Lee J. (2018). The complete plastome sequence of an Antarctic Bryophyte *Sanionia uncinata* (Hedw.) Loeske. Int. J. Mol. Sci..

[19] Saina J., Li Z.-Z., Gichira A.W., Liao Y.-Y. (2018). The complete chloroplast genome sequence of tree of heaven (*Ailanthus altissima* (Mill.) (Sapindales: Simaroubaceae), an important pantropical tree. Int. J. Mol. Sci..

[20] Xie D.-F., Yu Y., Deng Y.-Q., Li J., Liu H.-Y., Zhou S.-D., He X.-J. (2018). Comparative analysis of the chloroplast genomes of the Chinese endemic genus *Urophysa* and their contribution to chloroplast phylogeny and adaptive evolution. Int. J. Mol. Sci..

[21] Kawabe A., Nukii H., Furiahata H.Y. (2018). Exploring the history of chloroplast capture in *Arabis* using whole chloroplast genome sequencing. Int. J. Mol. Sci..

[22] Niu Z., Xue Q., Wang H., Xie X., Zhu S., Liu W., Ding X. (2017). Mutational biases and GC-biased gene conversion affect GC content in the plastomes of *Dendrobium* genus. Int. J. Mol. Sci..

[23] Lin G.-M., Lai Y.-H., Audira G., Hsiao C.-D. (2017). A simple method to decode the complete 18-5.8-28 rRNA repeated units of green algae by genome skimming. Int. J. Mol. Sci..

[24] Yin K., Zhang Y., Li Y., Du F.K. (2018). Different natural selection pressures on the *atpF* gene in evergreen sclerophyllous and deciduous oak species: Evidence from comparative analysis of the complete chloroplast genome of *Quercus aquifolioides* with other oak species. Int. J. Mol. Sci..

[25] Martín M., Sabater B. (1989). Translational control of chloroplast protein synthesis during senescence of barley leaves. Physiol. Plant..

[26] Del Campo E.M., Sabater B., Martín M. (2000). Transcripts of the ndhH-D operon of barley plastids: Possible role of unedited site III in splicing of the ndhA intron. Nucleic Acids Res..

[27] Wu H., Shi N., An X., Liu C., Fu H., Cao L., Feng Y., Sun D., Zhang L. (2018). Candidate genes for yellow leaf color in common wheat (*Triticum aestivum* L.) and major related metabolic pathways according to transcriptome profiling. Int. J. Mol. Sci..

[28] Legen J., Schmitz-Linneweber C. (2017). Stable membrane-association of mRNAs in etiolated, greening and mature plastids. Int. J. Mol. Sci..

[29] Vojta L., Culetic A., Fulgosi H. (2018). Effects of TROL presequence mutagenesis on its import and dual localization in chloroplasts. Int. J. Mol. Sci..

[30] Shao R., Zheng H., Jia S., Jiang Y., Yang Q., Kang G. (2018). Nitric oxide enhancing resistance to PEG-induced water deficiency is associated with the primary photosynthesis reaction in *Triticum aestivum* L.. Int. J. Mol. Sci..

[31] Wang Q.-L., Chen J.-H., He N.-Y., Guo F.-Q. (2018). Metabolic reprogramming in chloroplasts under heat stress in plants. Int. J. Mol. Sci..

[32] Izumi M., Nakamura S. (2018). Chloroplast protein turnover: The influence of extraplastidic processes, including autophagy. Int. J. Mol. Sci..

[33] Yoshida Y. (2018). Insights into the mechanisms of chloroplast division. Int. J. Mol. Sci..

[34] Ireda H., Shiomi D. (2018). Bacterial heterologous expression system for reconstitution of chloroplast inner division ring and evaluation of its contributors. Int. J. Mol. Sci..

[35] Suetsugu N., Wada M. (2017). Two coiled-coil proteins, WEB1 and PMI2, suppress the signalling pathway of chloroplast accumulation response that is mediated by two phototropin-interacting proteins, RPT2 and NCH1, in seed plants. Int. J. Mol. Sci..

[36] Halliwell B. (1983). Methods in chloroplast molecular biology. FEBS Lett..

